# Positive genetic interactors of *HMG2* identify a new set of genetic perturbations for improving sesquiterpene production in *Saccharomyces cerevisiae*

**DOI:** 10.1186/1475-2859-11-162

**Published:** 2012-12-22

**Authors:** Codruta Ignea, Fotini A Trikka, Ioannis Kourtzelis, Anagnostis Argiriou, Angelos K Kanellis, Sotirios C Kampranis, Antonios M Makris

**Affiliations:** 1 Centre International de Hautes Etudes Agronomiques Méditerranéennes, Mediterranean Agronomic Institute of Chania, P.O. Box 85, Chania, 73100, Greece; 2 Institute of Applied Biosciences/CERTH, P.O. Box 60361, Thermi, 57001, Thessaloniki, Greece; 3 School of Biology, Aristotle University of Thessaloniki, Thessaloniki, Greece; 4 Group of Biotechnology of Pharmaceutical Plants, Lab. of Pharmacognosy, Department of Pharmaceutical Sciences, Aristotle University of Thessaloniki, Thessaloniki, Greece; 5 Department of Medicine, University of Crete, P.O. Box 2208, Heraklion, 71003, Greece

## Abstract

**Background:**

Terpenoids and isoprenoids are an important class of natural products, which includes currently used drugs, high value bioactive and industrial compounds, and fuel candidates. Due to their industrial application, there is increasing interest in the development of *S. cerevisiae* strains capable of producing high levels of terpenoids.

**Results:**

Aiming to identify new gene targets which can be manipulated to increase sesquiterpene production, a set of *HMG2* positive genetic interactors were assessed as single and digenic heterozygous deletions in the presence or absence of stable *HMG2*(K6R) overexpression. Upon single allele deletion, most genes examined led to increased sesquiterpene production in yeast cells. Tandem heterozygous deletion of a set of three genes, the ubiquitin ligases *ubc7* and *ssm4/doa10*, and the ER resident protein *pho86*, led to an 11-fold increase in caryophyllene yields (125 mg/L in shake flasks) compared to cells lacking these modifications. The effect of the heterozygous deletions appears to be due to Hmg1p and Hmg2p stabilization.

**Conclusion:**

Heterozygous deletions cause significant reductions in protein levels but do not lead to growth impediments frequently seen in haploid strains. By exploiting desirable haploinsufficiencies in yeast, we identified a new set of genes that can be disrupted in tandem and cause significant stabilization of Hmgp and a substantial increase in sesquiterpene production. The approach presented here allows new genetic perturbations to be compiled on yeast cell factory strains without negatively impacting cell growth and viability.

## Background

*Saccharomyces cerevisiae* is a unicellular eukaryotic organism widely used as a cell factory for biotechnological applications. In recent years, strain improvements based on the availability of new genetic tools has accelerated the number of commercial applications of the organism. More than 12,400 patents using *S. cerevisiae* for the production of pharmaceuticals, food ingredients, chemicals and fuels have been filed [1]. Terpenoids and isoprenoids constitute an important class of secondary metabolites that contributes more than 50,000 compounds [2] to the rich chemical diversity of natural product structures. They include drugs, high-value bioactive and industrial compounds, and fuel candidates. All terpenoids are biosynthesized from two C_5_ precursors, isopentenyl diphosphate (IPP) and dimethylallyl diphosphate (DMAPP) [3]. Two distinct and independent biosynthetic routes to IPP formation exist. In yeast and mammals IPP originates from acetyl-CoA through the intermediate mevalonic acid (MVA). HMG-CoA reductase is a key enzyme of the MVA pathway. Yeast has two HMGR isozymes, Hmg1p and Hmg2p, which share a similar overall structure composed of an N-terminal anchor domain spanning the ER-membrane eight times, followed by a linker and a C-terminal catalytic domain. Genetic analysis have identified Ubiquitin ligase components (*HRD1* and *HRD3*), which function alongside a distinct Endoplasmic Reticulum Associated Degradation (ERAD) pathway, to be associated with regulated degradation of Hmgp [4-6]. The Doa10p (*SSM4*) Ubiquitin ligase plays a central role in the ERAD pathway by mediating the degradation of a variety of misfolded ER proteins.

A recent study uncovered the genetic landscape of a yeast cell by studying the effects of millions of digenic deletions on cell fitness [7]. Digenic deletions with *HMG2* identified a group of genes whose simultaneous deletion alleviates the growth impediments of *hmg2* deletion. This group constitutes the positive genetic interactors. Reasoning that under conditions of high terpene precursor production an increasing amount of stress will be imposed on *HMG2*, deletions of some of these genes may help alleviate such stress. Notably, a subset of the *hmg2* positive genetic interactors belongs to the ERAD complex involved in the regulation of Hmg1p and Hmg2p stability. Aiming to capitalize on desirable haploinsufficiencies so as to achieve increased terpene substrate (FPP) formation, a set of positive genetic interactors was tested as diploid heterozygous deletion strains to identify genes which could be perturbed in tandem to improve a terpene production platform strain. Heterozygous deletions of three such genes *ubc7, ssm4* and *pho86* led to an 11-fold increase in caryophyllene production over the basis strain and 50-fold improvement when compared to wild type yeast cells.

## Results

### Selection of candidate genes for perturbation

**Figure 1:**
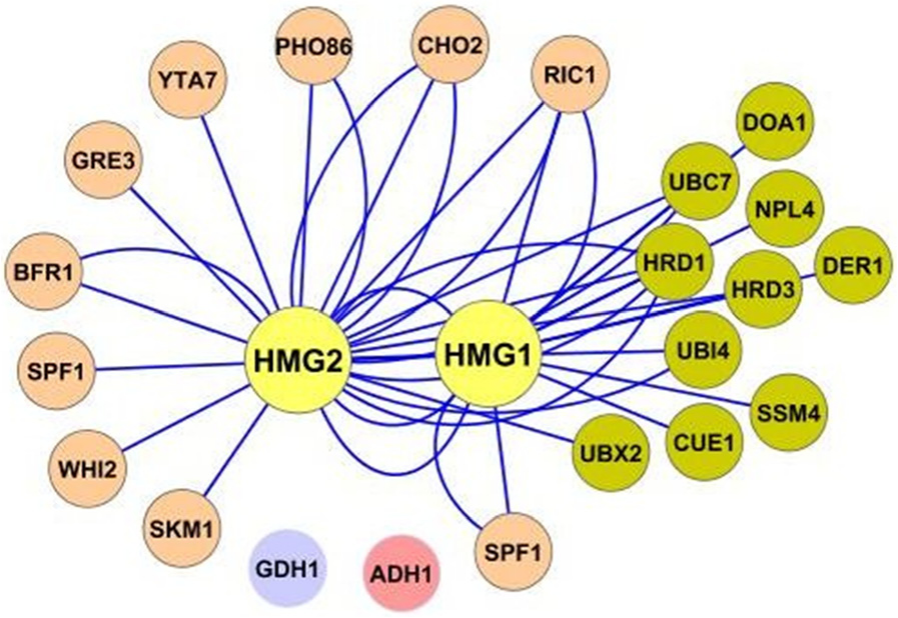
**Positive genetic interactors to HMG2 and HMG1 genes were selected for testing their contribution to increased terpenoid production in *S. cerevisiae *cells.** In dark green color are shown members of the ER-Associated protein Degradation pathway (ERAD), involved in recognition and ubiquitination of ER proteins destined for degradation. GDH1 was previously identified as target for improvement of sesquiterpene biosynthesis in yeast. ADH1 is used as control. The diagram was drawn using Cytoscape open software [12].

In previous work for the development of optimized *S. cerevisiae* strains producing higher levels of monoterpenoids and sesquiterpenoids [8] we observed that a strategy based on heterozygous gene deletions, taking advantage of gene expression reduction and haploinsufficiencies, can be used to improve terpene production through suppression of competing genes (*ERG9*) [8,9]. To capitalize on this observation, we undertook to identify and assess a series of single allele deletions which could contribute to further increasing sesquiterpene yields. A group of yeast strains deleted for genes previously identified as *HMG2* positive genetic interactors was selected, reasoning that under conditions of high terpene precursor production which impose stress on the MVA pathway, cells would be stressed in a similar manner to *hmg2* deletion, and their deletion could alleviate such stress (Figure 1). Positive *HMG2* genetic interactors represent gene deletions which compensate growth impediments caused by *hmg2* deletion. Interestingly, a large subset of these positive genetic interactors belongs to the Endoplasmic Reticulum Associated Degradation complex (ERAD, shown in dark green in Figure 1), which is involved in the degradation of ER transmembrane proteins, such as hmg1p and hmg2p [6,10]. To this group, we added *GDH1* as positive control, since it was previously shown that *gdh1* cells produce 85% higher levels of the sesquiterpene cubebol than parental cells [11], and *ADH1* as negative control.

### Sesquiterpene production in heterozygous deletion yeast strains

**Figure 2:**
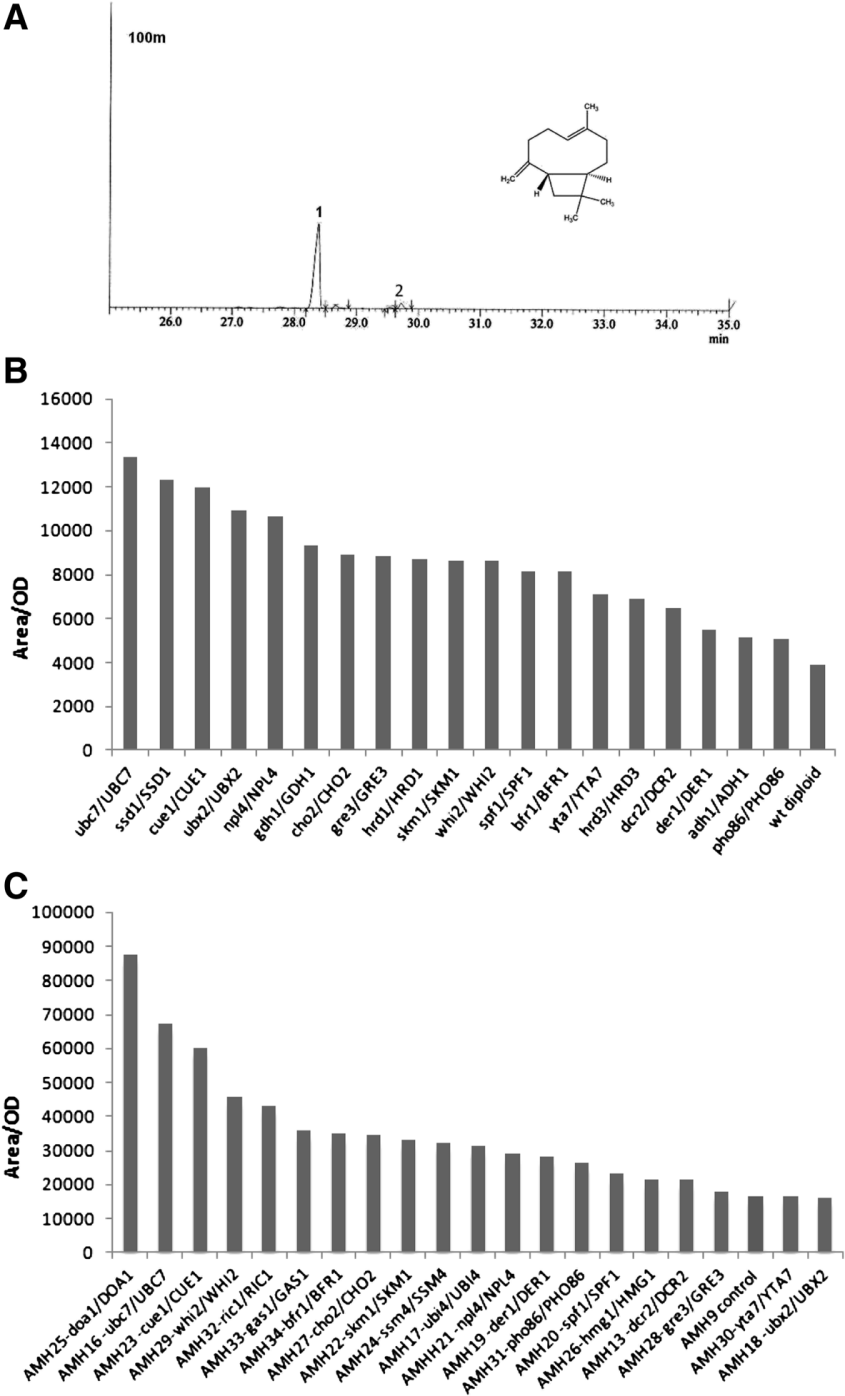
**Caryophyllene production in heterozygous deletion strains.** (**A**) Expression of Sf126 orf in yeast yields trans-caryophyllene (peak 1) and minor amounts of α-humulene (peak 2) sampled by SPME and analyzed by GC-MS; (**B**) production of caryophyllene by diploid heterozygous deletion strains in BY4743 genetic background. BY4743 cells were used as control strains; (**C**) production of caryophyllene in diploid heterozygous deletion strains overexpressing stable variants of HMG2 (K6R) from extra gene copies integrated into the chromosomes.

**Figure 3:**
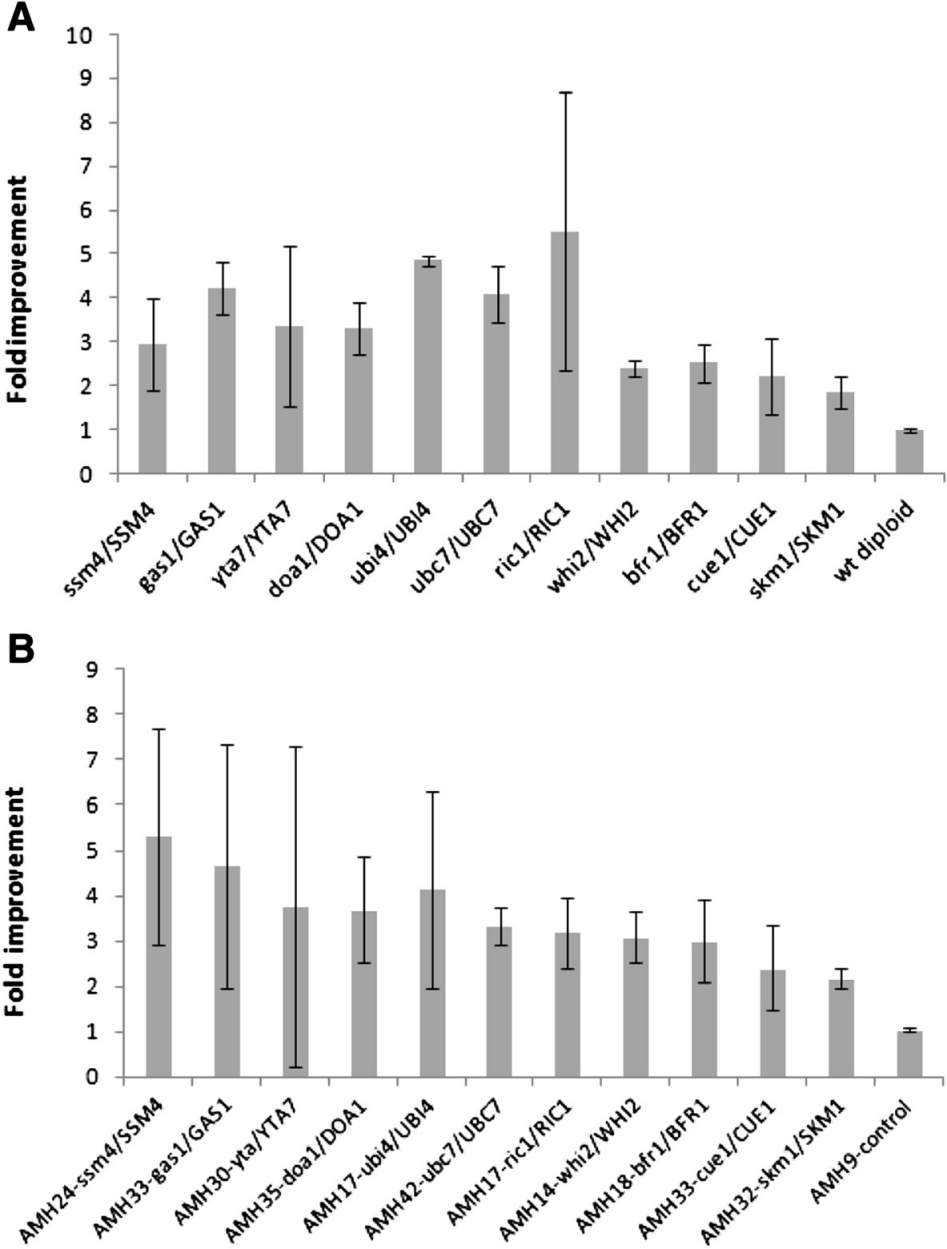
**Caryophyllene increase in selected strains which were analyzed over multiple independent experiments, in the course of one month period.** (**A**) Selected diploid heterozygous deletion strains in BY4743 wild type genetic background; (**B**) diploid heterozygous deletion strains in genetic background overexpressing HMG2 (K6R).

To assess the effect of the deletion of the selected genes in sesquiterpene production, these were tested as heterozygous deletions in diploid cells. Deletion heterozygocity is expected to lead to almost 50% reduction in expressed gene levels [13]. To assess the effect of the heterozygous deletions in sesquiterpene product yield, we chose to express the *Salvia fruticosa* (Greek sage) Sf126 cDNA which encodes for a sesquiterpene synthase producing >90% trans-*β*-caryophyllene, with minor amounts of *α*-humulene (Figure 2A). Wild type Mat *α* cells carrying the pUTDH3/Sf126 plasmid expressing the caryophyllene synthase under the control of strong glycolytic P_TDH3_ promoter were mated to the selected set of BY4741 gene deletion mutants, generating a set of heterozygous deletion diploid strains (Table 1, AMW2-25 strains). Each strain was grown in 50 ml flask cultures to saturation, the ambient volatiles were sampled by Solid Phase Micro Extraction (SPME), and the adsorbed compounds were analyzed by Gas chromatography–mass spectrometry (GC-MS). The relative caryophyllene yields were normalized for culture density and the results were compared to wild type diploid cells of the same genetic background (Figure 2B). All examined strains exhibited higher production of caryophyllene compared to control cells. Two thirds of the strains showed greater than two-fold increase (*ubc7/UBC7, ssd1/SSD1, cue1/CUE1, ubx2/UBX2, npl4/NPL4, gdh1/GDH1, cho2/CHO2, gre3/GRE3, hrd1/HRD1, skm1/SKM1, whi2/WHI2, spf1/SPF1, bfr1/BFR1*). To test whether the same heterozygous deletions could confer a production advantage in a strain overexpressing hmg2p, the AM88-01 Mat *α* strain (Mat α, P_Gal1_*HMG2*(K6R)::HO, *ura3, trp1*, P_TDH3_*HMG2*(K6R)-HIS5::*leu2)* expressing a chromosomal integrated copy of a stable *HMG2* variant (K6R) and Sf126 from the plasmid, was mated to the same set of BY4741 deletion mutants as above, generating strains AMH13 to AMH34 (Table 1). Consistent with previous findings, analysis of caryophyllene production showed higher overall production levels due to hmg2(K6R)p overexpression (Figure 2C). Still, the heterozygous deletions appear to also function in this genetic background, conferring an additional production boost, which could reach up to 5-fold higher production compared to AMH9 control cells (AMH25 strain *doa1/DOA1*, Figure 2C). When AMH25 cells were compared to wild type diploid cells derived from a cross between EG60 and BY4741 improvement exceeded 20-fold. To assess the reproducibility of caryophyllene production with time, we tested a smaller selected set of strains in replicate experiments over a period of one month. The analyzed strains reproducibly showed high levels of caryophyllene production. Only the *yta7/YTA7* heterozygous deletion exhibited instability, with yield reductions over time in both genetic backgrounds (Figure 3A and B). Among the candidate genes for sesquiterpene increase (*ssm4*, *gas1*, *doa1*, *ubi4*, *ubc7*, *ric1*, *whi2*, *cue1*, *skm1*), the *ubc7* deletion was selected as the first target for modification, since *ubc7*/*UBC7* cells were highly stable caryophyllene overproducers in both genetic backgrounds tested (wild type and *HMG2* (K6R) overexpressing).

**Table 1:** **List of *S. cerevisiae *strains used**

**Strain**	**Genotype**	**Plasmid description**	**Source**
BY4741	Mat a, *his3*Δ*1, leu2*Δ*0, met15*Δ*0, ura3*Δ*0.*		Research Genetics
EG60	Mat α, *ura3, trp1, his3.*		E. Golemis
EG60-01	Mat α, *ura3, trp1, his3.*	pUTDH3/Sf126, 2 μ URA3 P_TDH3_-Sf126	This study
AM62	Mat α, *ura3, trp1, his3, ubc7*:: HIS5		This study
AM62-01	Mat α, *ura3, trp1, his3, ubc7*:: HIS5	pUTDH3/Sf126, 2 μ URA3 P_TDH3_-Sf126	This study
AM66	Mat α/a, P_Gal1_-*HMG2*(K6R)*x*2::HO, *ura3, trp1, his3*. Derivative of AM63 and KSY10 back-crossed 4 times.		This study
AM85	Mat α/a, P_Gal1_-*HMG2*(K6R)*x*2::HO, *ura3, trp1*, TDH3p-*HMG2*(K6R)-HIS3::*leu2*. Derivative of AM66		This study
AM88	Mat α, P_Gal1_-*HMG2*(K6R)::HO, *ura3, trp1*, P_TDH3_-*HMG2*(K6R)-HIS3::*leu2*. Derived from sporulation of AM85 and subsequent excision of HIS3		This study
AM88-01	Mat α, P_Gal1_-*HMG2*(K6R)::HO, *ura3, trp1*, P_TDH3_-*HMG2*(K6R)-HIS3::*leu2*. Derived from sporulation of AM85 and subsequent excision of HIS3	pUTDH3/Sf126, 2 μ URA3 P_TDH3_-Sf126	This study
AM89	Mat a, P_Gal1_-*HMG2*(K6R)::HO, *ura3, trp1*, P_TDH3_-*HMG2*(K6R)-HIS3::leu2. Derived from sporulation of AM85 and subsequent excision of HIS3		This study
AM90	Mat a/α, P_Gal1_-*HMG2*(K6R):: HOX2, *ura3, trp1*, *his3*, P_TDH3_-*HMG2*(K6R)::*leu2 X*2. Derived from cross of AM88 and AM89		This study
AM94	Mat a/α, P_Gal1_-*HMG2*(K6R):: HOX2, *ura3, trp1*, *his3,* P_TDH3_-*HMG2*(K6R)::*leu2 X*2, ERG9/*erg9* Derived from AM90		This study
AM94-01	Mat a/α, P_Gal1_-HMG2(K6R):: HOX2, *ura3, trp1*, *his3,* P_TDH3_-*HMG2*(K6R)::*leu2 X*2, *ERG9*/*erg9* Derived from AM90	pUTDH3/Sf126, 2 μ URA3 P_TDH3_-Sf126	This study
AM97	Mat a/α, P_Gal1_-*HMG2*(K6R):: HOX2, *ura3, trp1*, *his3,* P_TDH3_-*HMG2*(K6R)::*leu2 X*2, *ERG9*/*erg9*, *UBC7*/*ubc7* derived from AM94		This study
AM97-01	Mat a/α, P_Gal1_-*HMG2*(K6R):: HOX2, *ura3, trp1*, *his3,* P_TDH3_-HMG2(K6R)*X*2-::*leu2* ERG9/*erg9*, UBC7/*ubc7* derived from AM94	pUTDH3/Sf126, 2 μ URA3 P_TDH3_-Sf126	This study
AM102	Mat a/α, P_Gal1_-*HMG2*(K6R):: HOX2, *ura3, trp1*, *his3,* P_TDH3_-*HMG2*(K6R)*X*2-::*leu2 ERG9*/*erg9*, *UBC7*/*ubc7*, *SSM4*/*ssm4* derived from AM97		This study
AM102-01	Mat a/α, P_Gal1_-*HMG2*(K6R):: HOX2, *ura3, trp1*, *his3,* P_TDH3_-*HMG2*(K6R)::*leu2 X*2, *ERG9*/*erg9*, *UBC7*/*ubc7*, *SSM4*/*ssm4* derived from AM97	pUTDH3/Sf126, 2 μ URA3 P_TDH3_-Sf126	This study
AM109	Mat a/α, P_Gal1_-*HMG2*(K6R):: HOX2, *ura3, trp1*, *his3,* P_TDH3_-*HMG2*(K6R)*X*2-::*leu2 ERG9*/*erg9*, *UBC7*/*ubc7*, *SSM4*/*ssm4, PHO86/pho86,* derived from AM102		This study
AM109-01	Mat a/α, P_Gal1_-*HMG2*(K6R)*x*2:: HOX2, *ura3, trp1*, *his3,* P_TDH3_-*HMG2*(K6R)*X*2-::*leu2 ERG9*/*erg9*, *UBC7*/*ubc7*, *SSM4*/*ssm4, PHO86/pho86,* derived from AM102	pUTDH3/Sf126, 2 μ URA3 P_TDH3_-Sf126	This study
AMW2	Mat a/α, *ura3, his3, leu2 TRP1*/*trp1*, *DCR2*/*dcr2::*G418	pUTDH3/Sf126, 2 μ URA3 P_TDH3_-Sf126	This study
AMW3	Mat a/α, *ura3, his3, leu2 TRP1*/*trp1*, *SSD1*/*ssd1::*G418	pUTDH3/Sf126, 2 μ URA3 P_TDH3_-Sf126	This study
AMW4	Mat a/α, *ura3, his3, leu2 TRP1*/*trp1*, *UBC7*/*ubc7::*G418	pUTDH3/Sf126, 2 μ URA3 P_TDH3_-Sf126	This study
AMW5	Mat a/α, *ura3, his3, leu2 TRP1*/*trp1*, *UBI4*/*ubi4::*G418	pUTDH3/Sf126, 2 μ URA3 P_TDH3_-Sf126	This study
AMW6	Mat a/α, *ura3, his3, leu2 TRP1*/*trp1*, *UBX2*/*ubx2::*G418	pUTDH3/Sf126, 2 μ URA3 P_TDH3_-Sf126	This study
AMW7	Mat a/α, *ura3, his3, leu2 TRP1*/*trp1*, *DER1*/*der1::*G418	pUTDH3/Sf126, 2 μ URA3 P_TDH3_-Sf126	This study
AMW8	Mat a/α, *ura3, his3, leu2 TRP1*/*trp1*, *SPF1*/*spf1::*G418	pUTDH3/Sf126, 2 μ URA3 P_TDH3_-Sf126	This study
AMW9	Mat a/α, *ura3, his3, leu2 TRP1*/*trp1*, *NPL4*/*npl4::*G418	pUTDH3/Sf126, 2 μ URA3 P_TDH3_-Sf126	This study
AMW10	Mat a/α, *ura3, his3, leu2 TRP1*/*trp1*, *SKM1*/*skm1::*G418	pUTDH3/Sf126, 2 μ URA3 P_TDH3_-Sf126	This study
AMW11	Mat a/α, *ura3, his3, leu2 TRP1*/*trp1*, *CUE1*/*cue1::*G418	pUTDH3/Sf126, 2 μ URA3 P_TDH3_-Sf126	This study
AMW12	Mat a/α, *ura3, his3, leu2 TRP1*/*trp1*, *SSM4*/*ssm4::*G418	pUTDH3/Sf126, 2 μ URA3 P_TDH3_-Sf126	This study
AMW13	Mat a/α, *ura3, his3, leu2 TRP1*/*trp1*, *DOA1*/*doa1::*G418	pUTDH3/Sf126, 2 μ URA3 P_TDH3_-Sf126	This study
AMW14	Mat a/α, *ura3, his3, leu2 TRP1*/*trp1*, *HMG1*/*hmg1::*G418	pUTDH3/Sf126, 2 μ URA3 P_TDH3_-Sf126	This study
AMW15	Mat a/α, *ura3, his3, leu2 TRP1*/*trp1*, *CHO2*/*cho2::*G418	pUTDH3/Sf126, 2 μ URA3 P_TDH3_-Sf126	This study
AMW16	Mat a/α, *ura3, his3, leu2 TRP1*/*trp1*, *GRE3*/*gre3::*G418	pUTDH3/Sf126, 2 μ URA3 P_TDH3_-Sf126	This study
AMW17	Mat a/α, *ura3, his3, leu2 TRP1*/*trp1*, *WHI2*/*whi2::*G418	pUTDH3/Sf126, 2 μ URA3 P_TDH3_-Sf126	This study
AMW18	Mat a/α, *ura3, his3, leu2 TRP1/trp1*, *YTA7*/*yta7::*G418	pUTDH3/Sf126, 2 μ URA3 P_TDH3_-Sf126	This study
AMW19	Mat a/α, *ura3, his3, leu2 TRP1*/*trp1*, *PHO86*/*pho86::*G418	pUTDH3/Sf126, 2 μ URA3 P_TDH3_-Sf126	This study
AMW20	Mat a/α, *ura3, his3, leu2 TRP1*/*trp1*, *RIC1*/*ric1::*G418	pUTDH3/Sf126, 2 μ URA3 P_TDH3_-Sf126	This study
AMW21	Mat a/α, *ura3, his3, leu2 TRP1*/*trp1*, *GAS1*/gas1*::*G418	pUTDH3/Sf126, 2 μ URA3 P_TDH3_-Sf126	This study
AMW22	Mat a/α, *ura3, his3, leu2 TRP1*/*trp1*, *ADH1*/*adh1::*G418	pUTDH3/Sf126, 2 μ URA3 P_TDH3_-Sf126	This study
AMW23	Mat a/α, *ura3, his3, leu2 TRP1*/*trp1*, *GDH1*/*gdh1*::G418	pUTDH3/Sf126, 2 μ URA3 P_TDH3_-Sf126	This study
AMW24	Mat a/α, *ura3, his3, leu2 TRP1*/*trp1*, *HRD1*/*hrd1*::G418	pUTDH3/Sf126, 2 μ URA3 P_TDH3_-Sf126	This study
AMW1	Mat a/α, *ura3, his3, leu2 TRP1*/*trp1*	pUTDH3/Sf126, 2 μ URA3 P_TDH3_-Sf126	This study
AMW25	Mat a/α, *ura3, his3, leu2 TRP1*/*trp1*, *HRD3*/*hrd3::*G418	pUTDH3/Sf126, 2 μ URA3 P_TDH3_-Sf126	This study
ΑΜΗ9	Mat a/α, P_Gal1_-*HMG2*(K6R)::HO*, ura3, his3, trp1*/*TRP1*, P_TDH3_-*HMG2*(K6R)-HIS5::*leu2*	pUTDH3/Sf126, 2 μ URA3 P_TDH3_-Sf126	This study
AMH13	Mat a/α, P_Gal1_-*HMG2*(K6R)::HO*, ura3, his3, trp1*/*TRP1*, P_TDH3_-*HMG2*(K6R)-HIS5::*leu2*, *DCR2*/*dcr2*::G418	pUTDH3/Sf126, 2 μ URA3 P_TDH3_-Sf126	This study
AMH15	Mat a/α, P_Gal1_-*HMG2*(K6R)::HO*, ura3, his3, trp1*/*TRP1*, P_TDH3_-*HMG2*(K6R)-HIS5::*leu2*, *SSD1*/*ssd1*::G418	pUTDH3/Sf126, 2 μ URA3 P_TDH3_-Sf126	This study
AMH16	Mat a/α, P_Gal1_-*HMG2*(K6R)::HO*, ura3, his3, trp1*/*TRP1*, P_TDH3_-*HMG2*(K6R)-HIS5::*leu2*, *UBC7*/*ubc7*::G418	pUTDH3/Sf126, 2 μ URA3 P_TDH3_-Sf126	This study
AMH17	Mat a/α, P_Gal1_-*HMG2*(K6R)::HO*, ura3, his3, trp1*/*TRP1*, P_TDH3_-*HMG2*(K6R)-HIS5::*leu2*, *UBI4*/*ubi4*::G418	pUTDH3/Sf126, 2 μ URA3 P_TDH3_-Sf126	This study
AMH18	Mat a/α, P_Gal1_-*HMG2*(K6R)::HO*, ura3, his3, trp1*/*TRP1*, P_TDH3_-*HMG2*(K6R)-HIS5::*leu2*, *UBX2/ubx2*::G418	pUTDH3/Sf126, 2 μ URA3 P_TDH3_-Sf126	This study
AMH19	Mat a/α, P_Gal1_-*HMG2*(K6R)::HO*, ura3, his3, trp1*/*TRP1*, P_TDH3_-*HMG2*(K6R)-HIS5::*leu2*, *DER1/der1*::G418	pUTDH3/Sf126, 2 μ URA3 P_TDH3_-Sf126	This study
AMH20	Mat a/α, P_Gal1_-*HMG2*(K6R)::HO*, ura3, his3, trp1*/*TRP1*, P_TDH3_-*HMG2*(K6R)-HIS3::*leu2*, *SPF1/spf1*::G418	pUTDH3/Sf126, 2 μ URA3 P_TDH3_-Sf126	This study
AMH21	Mat a/α, P_Gal1_-*HMG2*(K6R)::HO*, ura3, his3, trp1*/*TRP1*, P_TDH3_-*HMG2*(K6R)-HIS5::*leu2*, *NPL4/npl4*::G418	pUTDH3/Sf126, 2 μ URA3 P_TDH3_-Sf126	This study
AMH22	Mat a/α, P_Gal1_-*HMG2*(K6R)::HO*, ura3, his3, trp1*/*TRP1*, P_TDH3_-*HMG2*(K6R)-HIS5::*leu2*, *SKM1*/*skm1*::G418	pUTDH3/Sf126, 2 μ URA3 P_TDH3_-Sf126	This study
AMH23	Mat a/α, P_Gal1_-*HMG2*(K6R)::HO*, ura3, his3, trp1*/*TRP1*, P_TDH3_-*HMG2*(K6R)-HIS5::*leu2*, *CUE1*/*cue1*::G418	pUTDH3/Sf126, 2 μ URA3 P_TDH3_-Sf126	This study
AMH24	Mat a/α, P_Gal1_-*HMG2*(K6R)::HO*, ura3, his3, trp1*/*TRP1*, P_TDH3_-*HMG2*(K6R)-HIS5::*leu2*, *SSM4*/*ssm4*::G418	pUTDH3/Sf126, 2 μ URA3 P_TDH3_-Sf126	This study
AMH25	Mat a/α, P_Gal1_-*HMG2*(K6R)::HO*, ura3, his3, trp1*/*TRP1*, P_TDH3_-*HMG2*(K6R)-HIS5::*leu2*, *DOA1/doa1*::G418	pUTDH3/Sf126, 2 μ URA3 P_TDH3_-Sf126	This study
AMH26	Mat a/α, P_Gal1_-*HMG2*(K6R)::HO*, ura3, his3, trp1*/*TRP1*, P_TDH3_-*HMG2*(K6R)-HIS5::*leu2*, *HMG1/hmg1*::G418	pUTDH3/Sf126, 2 μ URA3 P_TDH3_-Sf126	This study
AMH27	Mat a/α, P_Gal1_-*HMG2*(K6R)::HO*, ura3, his3, trp1*/*TRP1*, P_TDH3_-*HMG2*(K6R)-HIS3::*leu2*, *CHO2*/*cho2*::G418	pUTDH3/Sf126, 2 μ URA3 P_TDH3_-Sf126	This study
AMH28	Mat a/α, P_Gal1_-HMG2(K6R)::HO*, ura3, his3, trp1*/*TRP1*, P_TDH3_-*HMG2*(K6R)-HIS5::*leu2*, *GRE3*/*gre3*::G418	pUTDH3/Sf126, 2 μ URA3 P_TDH3_-Sf126	This study
AMH29	Mat a/α, P_Gal1_-*HMG2*(K6R)::HO*, ura3, his3, trp1*/*TRP1*, P_TDH3_-*HMG2*(K6R)-HIS5::*leu2*, *WHI2*/*whi2*::G418	pUTDH3/Sf126, 2 μ URA3 P_TDH3_-Sf126	This study
AMH30	Mat a/α, P_Gal1_-*HMG2*(K6R)::HO*, ura3, his3, trp1*/*TRP1*, P_TDH3_-*HMG2*(K6R)-HIS5::*leu2*, *YTA7/yta7*::G418	pUTDH3/Sf126, 2 μ URA3 P_TDH3_-Sf126	This study
AMH31	Mat a/α, P_Gal1_-*HMG2*(K6R)::HO*, ura3, his3, trp1*/*TRP1*, P_TDH3_-*HMG2*(K6R)-HIS5::*leu2*, *PHO86*/*pho86*::G418	pUTDH3/Sf126, 2 μ URA3 P_TDH3_-Sf126	This study
AMH32	Mat a/α, P_Gal1_-*HMG2*(K6R)::HO*, ura3, his3, trp1*/*TRP1*, P_TDH3_-*HMG2*(K6R)-HIS5::*leu2*, *RIC1/ric1*::G418	pUTDH3/Sf126, 2 μ URA3 P_TDH3_-Sf126	This study
AMH33	Mat a/α, P_Gal1_-*HMG2*(K6R)::HO*, ura3, his3, trp1*/*TRP1*, P_TDH3_-*HMG2*(K6R)-HIS5::*leu2*, *GAS1/gas1*::G418	pUTDH3/Sf126, 2 μ URA3 P_TDH3_-Sf126	This study
AMH34	Mat a/α, P_Gal1_-*HMG2*(K6R)::HO*, ura3, his3, trp1*/*TRP1*, P_TDH3_-*HMG2*(K6R)-HIS5::*leu2*, *BFR1/bfr1*::G418	pUTDH3/Sf126, 2 μ URA3 P_TDH3_-Sf126	This study
AMH47	Mat a/α, *ura3, his3, leu2 TRP1*/*trp1*, *DCR2*/*dcr2::*G418, UBC7/*ubc7*:: HIS5	pUTDH3/Sf126, 2 μ URA3 P_TDH3_-Sf126	This study
AMH49	Mat a/α, *ura3, his3, leu2 TRP1*/*trp1*, *SSD1*/*ssd1::*G418, UBC7/*ubc7*:: HIS5	pUTDH3/Sf126, 2 μ URA3 P_TDH3_-Sf126	This study
AMH50	Mat a/α, *ura3, his3, leu2 TRP1*/*trp1*, *ubc7::*G418, *ubc7*:: HIS5	pUTDH3/Sf126, 2 μ URA3 P_TDH3_-Sf126	This study
AMH51	Mat a/α, *ura3, his3, leu2 TRP1*/*trp1*, *UBI4*/*ubi4::*G418, UBC7/ubc7:: HIS5	pUTDH3/Sf126, 2 μ URA3 P_TDH3_-Sf126	This study
AMH52	Mat a/α, *ura3, his3, leu2 TRP1*/*trp1*, *UBX2*/*ubx2::*G418, UBC7/*ubc7*:: HIS5	pUTDH3/Sf126, 2 μ URA3 P_TDH3_-Sf126	This study
AMH53	Mat a/α, *ura3, his3, leu2 TRP1*/*trp1*, *DER1*/*der1::*G418, *UBC7*/*ubc7*:: HIS5	pUTDH3/Sf126, 2 μ URA3 P_TDH3_-Sf126	This study
AMH54	Mat a/α, *ura3, his3, leu2 TRP1*/*trp1*, *SPF1*/*spf1::*G418, *UBC7*/*ubc7*:: HIS5	pUTDH3/Sf126, 2 μ URA3 P_TDH3_-Sf126	This study
AMH55	Mat a/α, *ura3, his3, leu2 TRP1*/*trp1*, *NPL4*/*npl4::*G418,*UBC7*/*ubc7*:: HIS5	pUTDH3/Sf126, 2 μ URA3 P_TDH3_-Sf126	This study
AMH56	Mat a/α, *ura3, his3, leu2 TRP1*/*trp1*, *SKM1*/*skm1::*G418, *UBC7*/*ubc7*:: HIS5	pUTDH3/Sf126, 2 μ URA3 P_TDH3_-Sf126	This study
AMH57	Mat a/α, *ura3, his3, leu2 TRP1*/*trp1*, *CUE1*/*cue1::*G418, *UBC7*/*ubc7*:: HIS5	pUTDH3/Sf126, 2 μ URA3 P_TDH3_-Sf126	This study
AMH58	Mat a/α, *ura3, his3, leu2 TRP1*/*trp1*, *SSM4*/*ssm4::*G418, *UBC7*/*ubc7*:: HIS5	pUTDH3/Sf126, 2 μ URA3 P_TDH3_-Sf126	This study
AMH59	Mat a/α, *ura3, his3, leu2 TRP1*/*trp1*, *DOA1*/*doa1::*G418, UBC7/*ubc7*:: HIS5	pUTDH3/Sf126, 2 μ URA3 P_TDH3_-Sf126	This study
AMH60	Mat a/α, *ura3, his3, leu2 TRP1*/*trp1*, *HMG1*/*hmg1::*G418, *UBC7*/*ubc7*:: HIS5	pUTDH3/Sf126, 2 μ URA3 P_TDH3_-Sf126	This study
AMH61	Mat a/α, *ura3, his3, leu2 TRP1*/*trp1*, *CHO2*/*cho2::*G418, *UBC7*/*ubc7*:: HIS5	pUTDH3/Sf126, 2 μ URA3 P_TDH3_-Sf126	This study
AMH62	Mat a/α, *ura3, his3, leu2 TRP1*/*trp1*, *GRE3*/*gre3::*G418, *UBC7*/*ubc7*:: HIS5	pUTDH3/Sf126, 2 μ URA3 P_TDH3_-Sf126	This study
AMH63	Mat a/α, *ura3, his3, leu2 TRP1*/*trp1*, *WHI2*/*whi2::*G418, *UBC7*/*ubc7*:: HIS5	pUTDH3/Sf126, 2 μ URA3 P_TDH3_-Sf126	This study
AMH64	Mat a/α, *ura3, his3, leu2 TRP1*/*trp1*, *YTA7*/*yta7::*G418, *UBC7*/*ubc7*:: HIS5	pUTDH3/Sf126, 2 μ URA3 P_TDH3_-Sf126	This study
AMH65	Mat a/α, *ura3, his3, leu2 TRP1*/*trp1*, *PHO86*/*pho86::*G418, *UBC7*/*ubc7*:: HIS5	pUTDH3/Sf126, 2 μ URA3 P_TDH3_-Sf126	This study
AMH66	Mat a/α, *ura3, his3, leu2 TRP1*/*trp1*, *RIC1/ric1::*G418, *UBC7*/*ubc7*:: HIS5	pUTDH3/Sf126, 2 μ URA3 P_TDH3_-Sf126	This study
AMH69	Mat a/α, *ura3, his3, leu2 TRP1*/*trp1*, *ADH1*/*adh1::*G418, *UBC7*/*ubc7*:: HIS5	pUTDH3/Sf126, 2 μ URA3 P_TDH3_-Sf126	This study
AMH70	Mat a/α, *ura3, his3, leu2 TRP1*/*trp1*, *GDH1*/*gdh1*::G418, *UBC7*/*ubc7*:: HIS5	pUTDH3/Sf126, 2 μ URA3 P_TDH3_-Sf126	This study
AMH74	Mat a/α, *ura3, his3, leu2 TRP1*/*trp1*, *HRD1*/*hrd1*::G418, *UBC7*/*ubc7*:: HIS5	pUTDH3/Sf126, 2 μ URA3 P_TDH3_-Sf126	This study
AMH76	Mat a/α, *ura3, his3, leu2 TRP1*/*trp1 UBC7*/*ubc7*:: HIS5 Control Strain	pUTDH3/Sf126, 2 μ URA3 P_TDH3_-Sf126	This study
AMH77	Mat a/α, *ura3, his3, leu2 TRP1*/*trp1 HRD3*/*hrd3::*G418, *UBC7*/*ubc7*:: HIS5	pUTDH3/Sf126, 2 μ URA3 P_TDH3_-Sf126	This study
AMH78	Mat a/alpha, ura3, his3, leu2 *TRP1*/trp1 *BFR1*/*bfr1::*G418, *UBC7*/*ubc7*:: HIS5	pUTDH3/Sf126, 2 μ URA3 P_TDH3_-Sf126	This study
AMD21	Mat α, *ura3, his3, leu2*, *pho86::*G418, *ubc7*:: HIS5	pUTDH3/Sf126, 2 μ URA3 P_TDH3_-Sf126	This study
AMD14	Mat α, *ura3, his3, leu2*, *ssm4::*G418, *ubc7*:: HIS5	pUTDH3/Sf126, 2 μ URA3 P_TDH3_-Sf126	This study
AMD5	Mat α, *ura3, his3, leu2*, *ssd1::*G418, *ubc7*:: HIS5	pUTDH3/Sf126, 2 μ URA3 P_TDH3_-Sf126	This study
AMD7	Mat α, *ura3, his3, leu2*, *ubi4::*G418, *ubc7*:: HIS5	pUTDH3/Sf126, 2 μ URA3 P_TDH3_-Sf126	This study
AMD33	Mat α, *ura3, his3, leu2*, *hrd3::*G418, *ubc7*:: HIS5	pUTDH3/Sf126, 2 μ URA3 P_TDH3_-Sf126	This study

**Figure 4:**
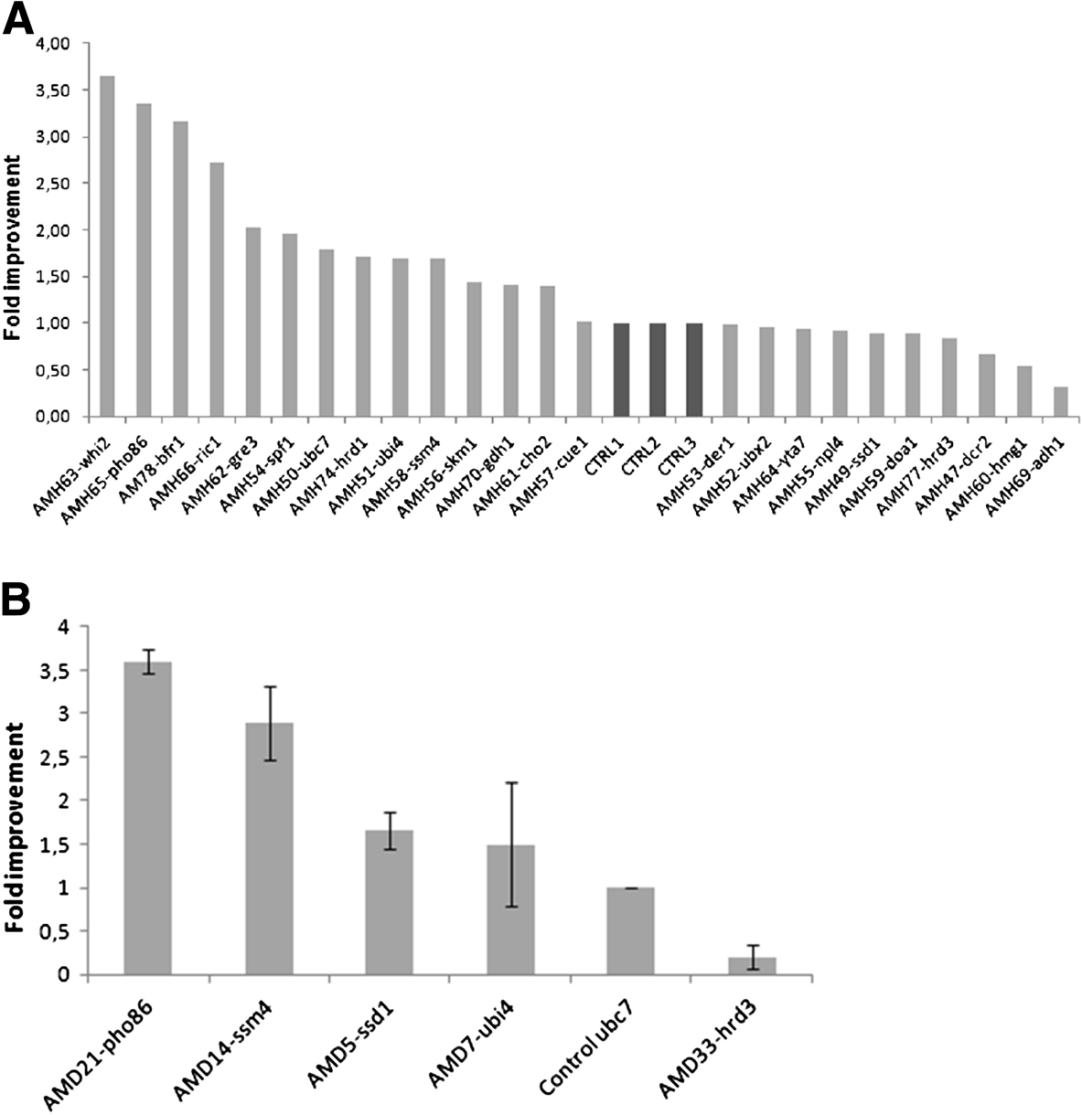
**Caryophyllene increase in diploid double heterozygous deletion strains.** (**A**) Caryophyllerne increase in strains harboring combinations of *ubc7* together with the selected genetic interactors. Independent cultures of *ubc7*/UBC7 cells were used as controls; (**B**) Caryophyllene increase in haploid double deletion strains for *ubc7* and selected genes which enhanced production compared to *ubc7* control cells.

To incorporate the *ubc7* deletion, Mat α wild type yeast cells were transformed with a cassette containing LoxP-HIS5-LoxP (pUG27) [14] flanked on each side by 50 bases which were homologous to the 5’ and 3’ end of *UBC7* respectively. The *UBC7* gene was excised by homologous recombination and the HIS growing colonies were selected and tested by PCR to verify gene deletion. The *ubc7*:: HIS5 strain AM62 carrying the pUTDH3/Sf126 plasmid, was mated to the selected BY4741 deletion mutants. Diploid strains were selected in glucose CM lacking uracil, histidine and tryptophan. The double heterozygous *ubc7*/*UBC7* (AMH47-78) diploid strains were assessed for caryophyllene production and were compared to single *ubc7*/*UBC7* heterozygous deletion strains used as controls. As seen in Figure 4A, a substantial number of heterozygous deletion pairs (*whi2, pho86, bfr1, ric1, spf1, hrd1, ubi4, ssm4, skm1, gdh1* and *cho2*) exhibit increased caryophyllene production over control cells. To finalize the set of deletions to be used jointly in a model production strain, the double homozygous deletion background was also tested in haploid cells. To construct them, the diploid strains were sporulated and the homozygous double deletion strains were identified for their capacity to grow in G418 in the absence of histidine and to mate with a haploid strain of the opposite mating type. The majority of double deletion strains performed significantly worse than the single *ubc7* deletion strain. Only the strains AMD21 (*ubc7*:: HIS5, *pho86*::G418), AMD14 (*ubc7*:: HIS5, *ssm4*::G418) and AMD5 (*ubc7*:: HIS5, ssm4::G418) performed better than the *ubc7*:: HIS5/*ubc7*::G418 control strain. All other strains fared worse. An approach based on deletion heterozygocity could yield strains that can be perturbed in more than two genes without losing fitness while suppression of essential genes could also be incorporated. Taking together these results, we decided to focus on *ubc7*, *ssm4* and *pho86* as the first triplet for heterozygous deletions in a yeast production strain.

### Development of an optimal yeast strain as basis for genetic perturbations to increase caryophyllene yields

**Figure 5:**
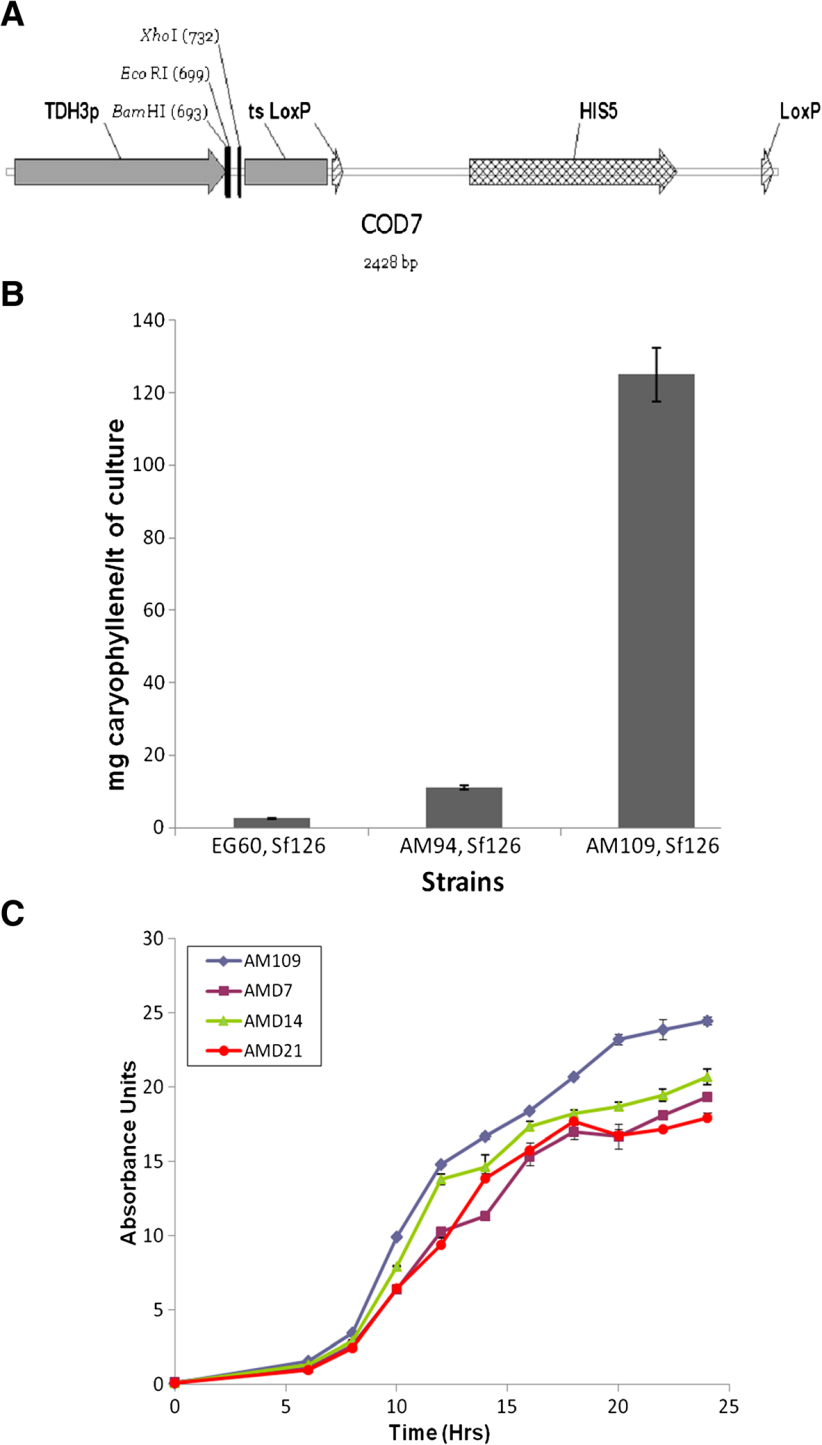
**Development of improved yeast strains for sesquiterpene production.** (**A**) Structure of the recyclable integration cassette COD7 used for integration of extra gene copies of HMG2 (K6R) in to desirable loci in the genome (Promoter P_TDH3_- cloning site-termination sequence-LoxP-HIS5-LoxP). Subsequent to integration of the cassette the HIS5 selection marker can be excised by the action of Cre recombinase; (**B**) Yields of caryophyllene measured after terpene extraction in EG60 wild type cells, AM94 cells which provided the basis for improvement and the final AM109 strain carrying *ubc7*, *ssm4(doa10)*, *pho86, erg9* heterozygous deletions; (**C**) Freshly grown AM109 (Mat a/α, P_Gal1_-*HMG2*(K6R):: HOX2, *ura3, trp1*, *his3,* P_TDH3_-*HMG2*(K6R)*X*2-::*leu2 ERG9*/*erg9*, *UBC7*/*ubc7*, *SSM4*/*ssm4, PHO86/pho86*), AMD7 (Mat α, *ura3, his3, leu2*, *ubi4::*G418, *ubc7*:: HIS5), AMD14 (Mat α, *ura3, his3, leu2*, *ssm4::*G418, *ubc7*:: HIS5) and AMD21 (Mat α, *ura3, his3, leu2*, *pho86::*G418, *ubc7*:: HIS5) cells were resuspended in fresh medium at the same OD_600_. The cultures were incubated shaking at 30°C. Samples were taken every 2 hours for a period of 24 hours and the OD_600_ was measured.

To establish an optimal strain for additional improvements, genetic changes previously identified as desirable for high terpene production were incorporated [8]. To switch the system towards constitutive terpene production, a new integration cassette was developed for stable integration of extra gene copies and expression under the strong P_TDH3_ promoter. The cassette named COD7 (Figure 5A) is composed of P_TDH3_-mcs-ts-LoxP-HIS5-LoxP. The *HMG2*(K6R) was inserted into the cloning site of COD7 and the construct was amplified by PCR with primers which introduce flanking sequences to the *leu2* gene. The transformed AM85 yeast strain exhibited higher terpene production (data not shown). To generate a diploid strain with 2 copies of P_TDH3_*HMG2*(K6R), AM85 cells were sporulated and haploid opposite mating type HIS strains were selected. The HIS5 module was excised using cre recombinase [8] and the generated strains were mated to develop AM90 strain. On this strain a single copy of *erg9* was deleted to generate AM94 cells (Mat a/α, P_Gal1_*HMG2*(K6R):: HOX2, *ura3, trp1*, *his3,* P_TDH3_*HMG2*(K6R)::*leu2 X*2, ERG9/*erg9*) which were the starting cell platform for further improvement (basis strain).

### Incorporation of identified gene deletions into AM94 cells

One of the *ubc7* alleles was deleted in AM94 cells generating the *ubc7*/*UBC7*, *erg9/ERG9* strain AM97. Subsequently, one allele of *ssm4* was deleted in AM97 cells generating the *ubc7/UBC7, ssm4/SSM4, erg9/ERG9* strain AM102. Finally, a single allele of *pho86* was deleted to generate strain AM109 (*ubc7/UBC7, ssm4/SSM4, pho86/PHO86, erg9/ERG9*).

To quantitatively assess caryophyllene production yields, cultures of EG60, AM94 and AM109 cells expressing Sf126 caryophyllene synthase were grown to saturation and caryophyllene was extracted. As seen in Figure 5B, AM109 cells produce 125 mg/L caryophyllene in shake flasks, which is 11-fold higher than AM94 cells (11.2 mg/L) and 50-fold higher than wild type haploid EG60 cells (2.5 mg/L). The results show that the heterozygous deletion combination strategy efficiently elevates sesquiterpene production in the improved yeast strains. To assess the growth properties of the AM109 strain harboring the heterozygous deletions we compared it with double homozygous deletions AMD7 (*ubi4, ubc7*), AMD14 (*ssm4, ubc7*) and AMD21 (*pho86, ubc7*), which were previously shown to exhibit increased improved caryophyllene production (Figure 4B). Cells were grown in glucose based media and growth was monitored by spectrophotometry at OD_600_. Dilutions of cells were measured when absorbance exceeded the linear scale. As seen in Figure 5C AM109 cells consistently grew faster and to higher saturation densities than all the homozygous double deletion AMD strains. AMD21 (*pho86, ubc7*) fared the worst, which is in accordance with previous observations that *PHO86* is a negative genetic interactor of *UBC7*. Quantitation of terpenoid yields by extraction yielded 6.1 mg/L for AMD7, 5.67 mg/L for AMD14, and 4.32 mg/L for AMD21 cells, which is >2-fold higher than wild type EG60 cells, but very low compared to the final engineered strain.

### Gene perturbations cause Hmgp stabilization

**Figure 6:**
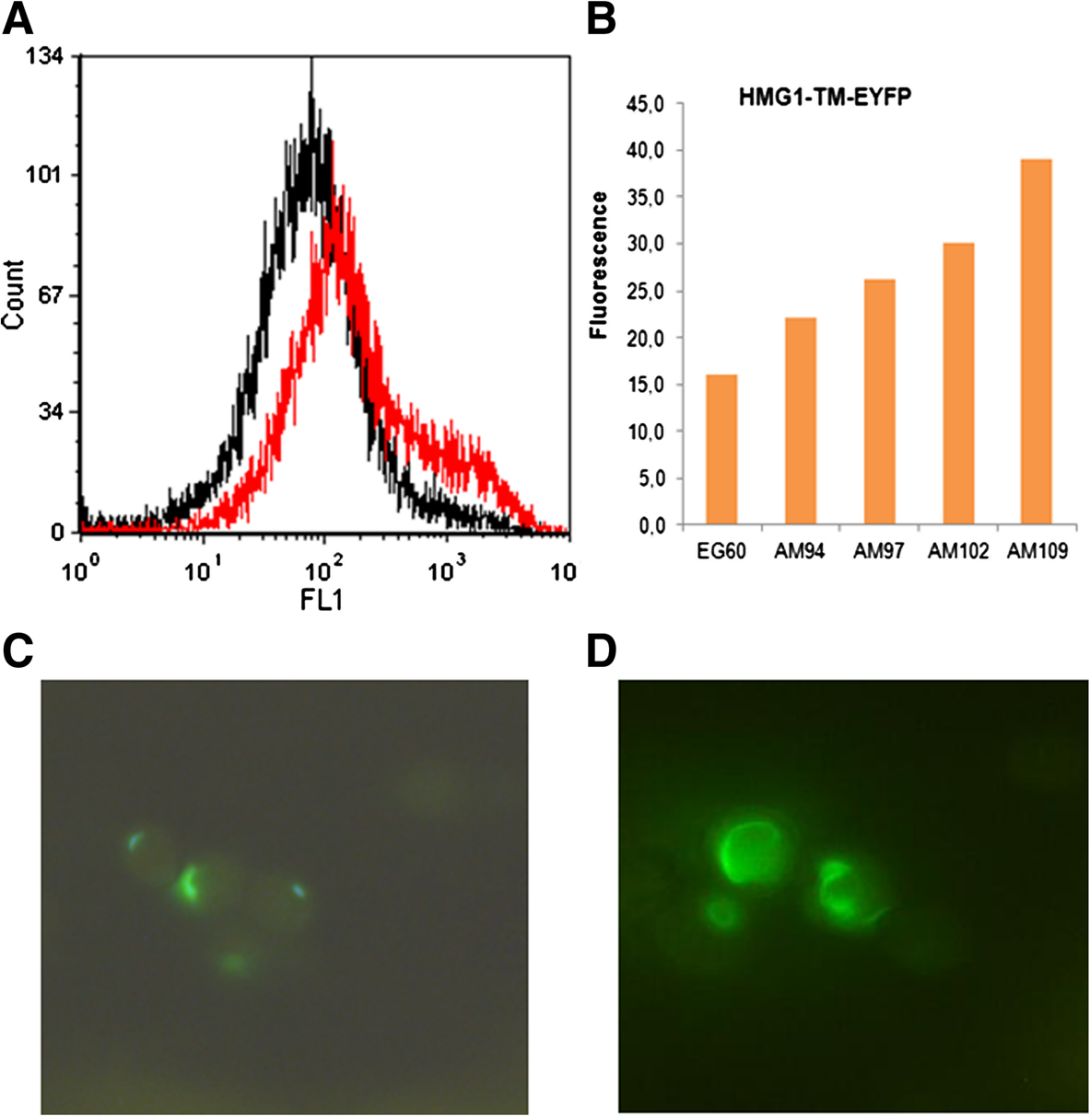
**Increased stabilization of hmg1p.** (**A**) Overlay of flow cytometry measurements of EG60 cells (black) and AM109 (red) overexpressing the transmembrane hmg1p domain fused to EYFP, hmg1-TM-EYFP, showing increased amounts of protein stability. Thirty thousand cells were counted; (**B**) Mean fluorescence values for strains analyzed show fluorescence increasing with each successive perturbation. (**C**) Hmg2-TM-EYFP fluorescing AM109 yeast cells; (**D**) Hmg1-TM-EYFP fluorescing AM109 yeast cells.

The identity of several of the gene deletions augmenting sesquiterpene production points to regulation of protein stability and degradation of the hmg1p and Hmg2p. To assess the effect of heterozygous deletions in hmgp stability, the transmembrane N-terminal 667 amino acids were fused to EYFP generating Hmg1p-TM-EYFP and Hmg2-TM-EYFP. The plasmid constructs were used to transform wild type EG60, AM94, AM97, AM102 and AM109 cells. Cells growing in logarithmic phase were examined by flow cytometry and fluorescent microscopy (Figure 6). The relative fluorescence of Hmg2-TM-EYFP was substantially less than that of hmg1-TM-EYFP in all strains tested (Figure 6C and D). In wild type cells hmg2-TM-EYFP was barely detectable. In AM109 cells the number of detectable fluorescing cells was higher and the intensity somewhat stronger (Figure 6C), but still at low level. The Hmg1-TM-EYFP fluorescence was significantly stronger in wild type cells and could easily be quantified by flow cytometry. Every genetic perturbation incorporated to the production strains led to higher numbers of fluorescing cells (Figure 6A and 6B). The mean fluorescence values of AM109 cells were almost 3-fold higher than wild type cells. The results show that the introduced heterozygous deletions enhance stability of hmgp which is considered a critical step in the MVA pathway for terpene substrate formation. No differences were observed in the intensity of fluorescence of an Sf126-EYFP fusion when expressed in the new strains indicating that the caryophyllene increase seen was not due to increased terpene synthase levels.

## Discussion

A recent study uncovered the genetic landscape of a yeast cell by studying the effects of millions of digenic deletions on cell fitness. Three types of genetic interactions were identified and quantified: a) negative interactions, where the second deletion causes more severe defects than expected, b) no interaction, where no deviation from the expected multiplicative effect is seen, and c) positive genetic interactions, where the second deletion alleviates the growth impediments of the first [7,15]. We selected a group of genes which were positive genetic interactors for *HMG2*, reasoning that in conditions of limited terpene precursor supply caused by the lower *HMG2* levels, deletion or downregulation of the positive interactors would alleviate the imposed stress by upregulating pathway components/regulators, releasing restrictions, or reducing competing activities.

A subset of the identified positive interactors belongs to the Endoplasmic Reticulum Associated Degradation complex (ERAD), a complex involved in the degradation of transmembrane proteins such as hmg2p and hmg1p [16,17]. Protein degradation by the ubiquitin-proteasome pathway is responsible for most cellular protein turnover. In yeast extensive research has focused on, regulated degradation of Hmg2p [6]. The conjugation of ubiquitin to proteins is a multistep process. Ub is first activated in an energy-dependent reaction by a Ub-activating enzyme (E1), and then transfered to a Ub-conjugating enzyme (E2). The E2 enzyme together with a third factor (E3), a Ubiquitin-ligase, transfer the Ub moiety to the identified substrate. The HRD complex of the ERAD machinery employs the Hrd1p E3 ligase, which, in conjunction with Hrd3p and a number of other proteins (Ubc7p, Cue1p, Der1p, Ubx2p, Npl4p, Cdc48p, Ufd1p, Usa1p, Yos9p), recognizes ER membrane and lumenal misfolded proteins, ubiquitinates them, and retrotranslocates them to the cytosolic side for protein degradation [6]. A second simpler complex named Doa10/Ssm4 recognizes cytosolic parts of misfolded proteins. It consists of Doa10p, Ubc7p, Cue1p, Ubx2p, and Ufd1p-Npl4p-Cdc48p [18,19]. Among the identified Hmgp positive genetic interactors, there are several members of the two ERAD complexes (*HRD1, HRD3, UBI4, UBX2, CUE1, DOA10/SSM4, DER1, UBC7, NPL4, DOA1*). All strains harboring heterozygous deletions in the above ERAD genes resulted in higher caryophyllene production. In the genetic background overexpressing the stable Hmg2p (K6R) variant, *ubx2/UBX2* could no longer contribute to terpene increases. The *ubc7*/*UBC7* deletion was chosen as the first target because of its capacity to reproducibly increase caryophyllene production over an extended time period tested. Moreover, the *ubc7* null mutation had previously been found to strongly inhibit Hmg2p ubiquitination and degradation [20]. When the *ubc7/UBC7* strains were tested as digenic heterozygous deletions with the other selected genes, most of the ERAD components (*cue1*/*CUE1*, *der1*/*DER1*, *ubx2*/*UBX2*, *npl4*/*NPL4*, *hrd3*/*HRD3*) offered no additional improvement. Only *hrd1*/*HRD1*, *ubi4*/*UBI4*, *ssm4*(*doa10*)/*SSM4*, or the homozygous deletion of *ubc7*/*ubc7* showed an improvement. This is not surprising as *UBI4* encodes for Ubiquitin, and Hrd1p appears to directly mediate the recognition of the integral membrane substrates [21]. The recognition capacity derives from the molecule’s transmembrane domain. Hrd1p employs two E2 ubiquitin-conjugating enzymes, Ubc7p and, to a lesser extent, Ubc1p [6]. These molecules are considered to be involved in the early events of ERAD function. Cue1p serves as an anchor for Ubc7p. However, Ubc7p may still bind to the Hrd complex in the absence of Cue1p. Derlin, Der1p, is believed to be involved in the movement of ER proteins across the ER membrane, while Ubx2p recruits the Cdc48p AAA-ATPase complex (Cdc48p/Npl4p/Ufd1p) to assist in the extraction of the proteins to the cytoplasm for proteasomal degradation [22]. These steps follow Hrd1p recognition-Ubc7p conjugation. The ssm4/doa10p complex attaches independently to Ubc7p via Cue1p. Through the action of Ubx2p, it recruits the Cdc48 complex [19]. Ssm4/Doa10p may independently recognize Hmg1p and Hmg2p, which could explain the observation that Ssm4/Doa10p suppression synergizes with Ubc7p haploinsufficiency.

For the remaining genes contributing to sesquiterpene production, there is limited information as to their mode of action. Pho86p is localized at the perinuclear compartment, as does Hmg2p. The protein has been implicated to function in the secretory pathway and may be required for the export of cargo molecules from the ER [23]. Our data indicate that deletion of *pho86*/*PHO86* increased Hmg1p stability. The *WHI2* gene plays a role in linking proliferation and cell response to environmental sensing mechanisms. Deletions in *whi2* cause defects in actin organization and apoptosis [24]. Homozygous *whi2/whi2* deletions, with or without *ubc7/ubc7* deletion, were detrimental to sesquiterpene productivity. However, *whi2*/*WHI2* strongly synergized with *ubc7*/*UBC7* to increase sesquiterpene productivity by >3.5-fold. A potential explanation for the effect of *WHI2* suppression may involve effects of the actin cytoskeleton in sensing osmotic stress. The osmotic stress regulating Hog1 MAP kinase pathway has been shown to repress ergosterol biosynthesis [25]. *SPF1/COD1* and *CHO2* were previously identified in a genetic screen for genes required for Hmg2p-induced ER remodeling [17]. Spf1/Cod1p is a multispanning ER transporter required for ER homeostasis. Although the gene was identified in an Hmg2p screen, microscopically the protein co-localizes with Hmg1p [17]. *CHO2* encodes for a phosphoethanolamine methyltransferase (PEMT). Overexpression of HMGR is known to induce a dramatic restructuring of ER membranes into highly organized arrays [17]. It is less clear though how ER remodeling affects the stability and function of HMGR.

The synergism of some of these heterologous deletions with perturbations of the ERAD pathway points to additional interventions that could further stabilize and maintain Hmgp in an active and stable state. This strategy can be complemented with perturbations affecting other facets of the biosynthetic pathway. One such target gene may be *GDH1*, which was recently identified by Asadollahi et al. [11] as a target for improvement. Deletion of the gene enhances cytosolic NADPH, the co-substrate of HMGR. In the context of this work, heterozygous deletion of *gdh1* contributed positively to caryophyllene production without any obvious growth impediments due to impairments in ammonia utilization.

## Conclusion

Positive genetic interactors to *HMG2* identified a new group of genes which upon heterozygous deletion can substantially enhance sesquiterpene production in yeast. Tandem deletions of three genes (*UBC7, SSM4/DOA10, PHO86*) led to an 11-fold increase in caryophyllene production over the unmodified basis strain. These desirable *HMG2* haploinsufficiencies can be compiled with additional perturbations at different critical points of the biosynthetic pathway. The strategy can be combined with complementary approaches such as gene overexpression, and fusion proteins of the metabolic pathway to reach maximal sesquiterpene yields.

## Methods

### Chemicals and materials

SPME fiber 2 cm-50/30um DVB/Carboxen™/PDMS StableFlex™ Fiber (Supelco); (−)-trans-caryophyllene (Sigma, C9653-5), was used as standard compound. Myc-Tag (9B11) mouse mAb, (#2276, Cell Signaling); and Anti-Mouse IgG (Fab specific)-Peroxidase antibody (Sigma, A9917) were used for protein detection. Superscript III Reverse Transcriptase (#18080-093, Invitrogen) was used for the construction of cDNA, MyTaq DNA polymerase (BIO-21105, Bioline), Accuzyme DNA polymerase (BIO-21051, Bioline) and Platinum Taq polymerase (#10966-018, Invitrogen) were used in PCR amplifications. Spectrum Plant Total RNA Kit 50 Prep (STRN50-1KT, Sigma) was used for plant RNA isolation. NucleoSpin Plasmid Kit (REF 740588.250, Macherey-Nagel) was used for plasmid DNA purification. QIAquick Gel Extraction Kit (#28704, Qiagen) was used for gel extraction and DNA purification. The sequences of oligonucleotide primers used is listed in Table 2.

**Table 2:** List of oligonucleotide primers used

**Primer name**	**Sequence**
5’TDH3p(HindIII)	5’-aagcttcagttcgagtttatcattatcaatactgcc-3’
3’TDH3p(BamHI)	5’- ggatccgtgtgtttattcgaaactaagttcttgg-3’
5’Sf126	5’-atggatattcctgtgattgttacttccgtttcg-3’
3’Sf126 XhoI	5’-ctcgagctagcaatcaagcatgaaggggtc-3’
5’GS linker	5’-gatcctatgtcgacggtagcggcagcggtagcggtagcggcagcgaattctatc-3’
3’GS linker	5’-tcgagatagaattcgctgccgctaccgctaccgctgccgctaccgtcgacatag-3’
5’Sf126	5’-atggatattcctgtgattgttacttccgtttcg-3’
HMG1-BamHI	5’-ggatccatgccgccgctattcaagggactgaaacagatggcaaa-3’
HMG1-TM-R-SalInS	5’-gtcgacgtcataatttttatatggtaaacgatcagatgctaatacagg-3’
5HMG2 EcoRI	5’-gaattcatgtcacttcccttaaaaacgatagtacat-3’
3HMG2-TM-XhoI	5’-ctcgagataatcataatttctgaagggcaa-3’
5’INT1-COD7	5’-gtttgccggtggtgcgaacaatagagcgaccatgaccttgaaggtgagacgcagttcgagtttatcattatc-3’
3’INT1-COD7	5’-ccagtgttgtatgtacctgtctatttatactggtagcaaccctggatctgatatcacctaataact-3’
ERG9-pUG F	5’-agagaaaagacgaagagcagaagcggaaaacgtatacacgtcacatatcacagctgaagcttcgtacgc-3’
ERG9-pUG R	5’-gtacttagttattgttcggagttgtttgtttatgttatttggcgcagactgcataggccactagtggatctg-3’
ERG9prom	5’-ctaaacgagcagcgagaacacgaccac-3’
UBC7 pUG F	5’-gagattatcctaaaaggaacttccctagtaatagtgtaatttggaagggcatagccagctgaagcttcgtacgc-3’
UBC7 pUG R	5’-gtatatagagaacagttaaaaggaagaccaaatgatcattaacctgctacctgctttcagcataggccactagtggatctg-3’
UBC7prom	5’-gaagaacttaccagactgtttcaagt-3’
SSM4-R	5’-tatatgtaaatatgctagcattcattttaaatgtaaggaagaaaacgcctgcataggccactagtggatctg-3’
SSM4-F	5’-tagccaagagtaccactaattgaatcaaagagactagaagtgtgaaagtccagctgaagcttcgtacgc-3’
SSM4prom	5’-gacattgaaaagatgattatgaccgatct-3’
PHO86-pUG F	5’-cgactcttccagttgcacattttttcatagcgaaagtacaagagtaggaacagctgaagcttcgtacgc-3’
PHO86-pUG R	5’-tttattttgttacttcctgcgagttgcaacagaactaacaagatgccatggcataggccactagtggatctg-3’

Yeast media: D (+)-Glucose monohydrate (16301, Sigma); Yeast Nitrogen Base w/o AA, carbohydrate & w/AS (Y2025, US Biologicals); Complete Minimal (CM) medium is composed of 0.13% (w/v) dropout powder (all essential amino acids), 0.67% (w/v) yeast nitrogen base w/o AA, 2% glucose; TOPO TA Cloning Kit Dual Promoter (K4610-20, Invitrogen); SuperSignal West Pico Chemiluminescent Substrate (34077, Thermo Scientific).

### Gene cloning and expression in yeast

The open reading frame of the Salvia fruticosa caryophyllene synthase Sf126 was amplified by high fidelity PCR (Accuzyme, BIOLINE). A’ overhangs were added to the purified PCR product and it was subsequently cloned to the pCRII vector by TOPO cloning. The clone orf in the desired orientation was digested with BamHI and XhoI and cloned into the pUTDH3 vector (P_TDH3_ 2 μ URA3) digested accordingly.

Construction of HMG1-TM-EYFP and HMG2-TM-EYFP. The N-terminal transmembrane domains of *HMG1* and *HMG2* spanning the first 667 amino acids were PCR amplified from yeast genomic DNA using primers HMG1-BamHI, HMG1-TM-R-SalInoS for HMG1 and 5HMG2EcoRI and 3HMG2-TM-XhoI for HMG2 respectively. The PCR products were cloned into PCR II vector as described above. The HMG1-TM domain was excised using BamHI and SalI restriction enzymes and ligated to the pUTDH3m/EYFP vector digested with BamHI and XhoI. The HMG2-TM domain was digested with EcoRI and SalI.

### Development of COD7 and COD70 cassette

The TDH3 promoter was PCR amplified using primers 5’TDH3p(HindIII) and 3’TDH3p(BamHI) which incorporate a HindIII and BamHI site at the 5’ and 3’ respectively. The PCR product was cloned by TOPO TA cloning into pCRII vector. The cassette vector COD4, previously described [8], was digested with HindIII and BamHI to excise the Gal1 promoter. The TDH3 promoter digested with the same enzymes was ligated into the plasmid cassette to generate COD7 (PTDH3-mcs-TS-LoxP-HIS3-LoxP). The variant HMG2 gene was cloned into COD7 using the EcoRI-XhoI restriction sites to generate the construct COD70 (TDH3p-*HMG2*(K6R)-TS-LoxP-HIS3-LoxP).

### Yeast strain development

The previously developed yeast strain AM66 [8] was transformed with the COD70 cassette amplified with primers 5’INT1-COD7 and 3’INT1-COD7, which incorporate flanking sequences complementary to the 5’ and 3’ of the *LEU2* gene respectively, to generate strain AM85. This diploid strain was sporulated and HIS positive haploid strains carrying the insertion were selected. Two opposite mating type HIS strains were transformed with plasmid pB227Gal-Cre and the HIS3 cassette was excised by the action of the expressed recombinase in Galactose-Raffinose based medium. The strains named AM88 and AM89 were crossed to generate diploid strain AM90 which carries two copies of the TDH3p-*HMG2*(K6R)-ts integron in the *leu2* locus. Subsequently, one of the two alleles of ERG9 was deleted using primers ERG9 pUG F primer and ERG9 pUG R primers as previously described [8]. Excision of the pUG72 URA3 cassette gave rise to strain AM94, which was the starting cell platform for the additional modifications. The single allele of UBC7 was deleted using primers UBC7 pUG F and UBC7 pUG R. Proper integration of the pUG72 cassette was verified by PCR from extracted genomic DNA using UBC7prom and UBC7 pUG R primers [14]. The cassette was subsequently excised giving rise to strain AM97. The UBC7 gene was also excised in EG60 wild type yeast cells using the pUG27 HIS5 LoxP cassette giving rise to strain AM62. To generate strain AM102, one of the two alleles of SSM4 gene was deleted. To this end, the pUG72 (LoxP-URA3-LoxP) cassette was amplified using primers SSM4-R and SSM4-F. The purified PCR cassette was used to transform AM97 cells. Integration was confirmed by PCR on genomic DNA from independent transformed colonies using primers SSMprom and SSM4-R. The URA3 marker was subsequently excised generating strain AM102. The final production strain AM109 was developed by deleting one allele of the *PHO86* gene on the AM102 genetic background. The pUG72 cassette was amplified using primers PHO86-pUG F and PHO86-pUG R. The purified PCR cassette was used to transform AM102 cells. Integration at the desired locus was confirmed by PCR on genomic DNA from independent transformed colonies using primers PHO86prom and PHO86-pUG R. Subsequent to URA3 excision, the generated strain AM109 was used for caryophyllene production.

### Construction of AMW yeast strains

A BY4742 yeast strain was previously rendered *trp1* by excising the wild type gene using a pUG LoxP cassette. The auxotrophic marker was excised as described above. The cells were transformed with plasmid pUTDH3/Sf126 carrying the caryophyllene synthase gene. The strain was crossed to selected deletion mutants from the BY4741 Research Genetics library. Diploid heterozygous deletion strains were selected in glucose Complete Media (CM) lacking tryptophan and uracil.

### Construction of AMH13-34 yeast strains

The Mat α AM88-01 strain harboring the pUTDH3/Sf126 plasmid was crossed to selected deletion mutants from the BY4741 Research Genetics library. Heterozygous diploid strains were selected in glucose CM media lacking tryptophan and uracil.

### Construction of AMH47-78 yeast strains

The AM62 Mat α strain carrying a deletion in *ubc7* which was substituted with the HIS5 gene, carrying the pUTDH3/Sf126 plasmid was mated to selected deletion mutants from the BY4741 Research Genetics library. The double heterozygous deletion strains were selected in glucose CM media lacking tryptophan, uracil and histidine.

### Construction of AMD strains

The double heterozygous deletion strains were sporulated. Haploid cells which were able to grow in G418, in the absence of uracil and histidine were selected out and mated with Mat a and Mat α reporter yeast strains to verify their mating type. Mat α haploid double knockouts carrying the pUTDH3/Sf126 plasmid were subsequently analyzed for caryophyllene production.

### Terpene extraction from yeast cells

Selected strains were cultivated in 50 ml liquid media containing 2% (wt/vol) Diaion HP20 (Supelco, Bellefonte, PA) as adsorbent resin. Prior to use, the resin was activated in 100% methanol. After 3 days incubation at 30°C, the beads were collected and washed with ddH_2_O to completely remove the yeast cells. The beads were then eluted three times with ethanol (30 min incubation), followed by three pentane elutions (30 min incubation). After addition of an equal volume of water and separation of the two phases, the pentane phase was concentrated to 100 μl final volume and analysed. The resulting main product was identified by GC–MS-based comparison to authentic standard, and relative amounts were quantified by GC-FID analysis.

### Flow cytometric studies

The plasmid constructs pUTDH3m/HMG1-TM-EYFP and pUTDH3m/HMG2-TM-EYFP were transformed to EG60, AM94, AM97, AM102 and AM109 cells. Overnight cultures of transformed cells were used to inoculate 50 ml cultures, and cells were grown to mid-log phase. Samples of cells were observed microscopically by fluorescent microscopy and 30,000 cells from each sample were measured for EYFP fluorescence by flow cytometry.

## Competing interests

The authors declare that they have no competing interests.

## Authors’ contributions

CI, FAT, IK conducted the experimental work. AA, AKK assisted in the data analysis, SCK assisted in experimental design, data analysis and review of the manuscript. AMM conducted some experiments and is responsible for the design and drafting of the paper. All authors read and approved the final manuscript.
